# Feasibility and Preliminary Efficacy of an Online Intervention to Increase Physical Activity in Nova Scotian Cancer Survivors: A Randomized Controlled Trial

**DOI:** 10.2196/cancer.4586

**Published:** 2015-11-23

**Authors:** Cynthia C Forbes, Chris M Blanchard, W Kerry Mummery, Kerry S Courneya

**Affiliations:** ^1^ University of Alberta Edmonton, AB Canada; ^2^ Dalhousie University Halifax, NS Canada

**Keywords:** Web-based, survivorship, home-based, exercise, efficacy, feasibility

## Abstract

**Background:**

Physical activity (PA) behavior change interventions among cancer survivors have used face-to-face, telephone, email, and print-based methods. However, computer-tailored, Internet-delivered programs may be a more viable option to achieve PA behavior change.

**Objective:**

The objective of this study is to test the feasibility and preliminary efficacy of a Web-based PA behavior change program among cancer survivors.

**Methods:**

Nova Scotian cancer survivors (N=415) who previously expressed interest in a research study were approached. Interested participants were asked to complete an online assessment of PA and quality of life (QOL) before being randomized to either a theory-based PA behavior change program using the PA tracking website UWALK (UCAN; n=48) or usual care (UC; n=47). After the intervention (9 weeks), participants completed another online assessment of PA and QOL as well as measures to evaluate the program and website. Descriptive analyses from surveys and Web analytic software were used to assess feasibility and mean change scores were used to test efficacy.

**Results:**

Of all contacted survivors, 95 (22.3%, 95/415) completed baseline measures and were randomized with 84 (88%, 84/95) completing the 9-week assessment. The behavior change program and website were rated highly on the satisfaction items. Average logins were 10.3 (1.1 per week) and 26.0% (111/432) of the weekly modules were completed. Most participants (71%, 29/41) indicated they were more aware of their daily PA levels and 68% (28/41) found the site easily navigable. Adjusted group differences in total exercise minutes favored the UCAN group by an increase of 42 minutes (95% CI -65 to 150; *P*=.44, d=0.17). Results were more pronounced, though still nonsignificant, among those not meeting guidelines at baseline where UCAN increased PA by 52 minutes compared to a decrease of 15 minutes in UC (adjusted between group difference=75, 95% CI -95 to 244; *P*=.38, d=0.27).

**Conclusions:**

We found that Internet-delivery may be a feasible alternative to more costly methods to promote PA among Nova Scotian cancer survivors. Moreover, there was a trend toward increased PA among those in the UCAN group, especially among those who were not meeting PA guidelines at baseline. Future research should focus on recruiting inactive cancer survivors and engaging them in the website to determine the optimal potential of Web-based interventions for promoting PA in cancer survivors.

## Introduction

Physical activity (PA) improves quality of life (QOL), symptom control, and possibly even survival in cancer survivors [1-8]. Despite these benefits, many cancer survivors do not accumulate the recommended 150 minutes of at least moderate-intensity PA per week [2,9,10]. A recent survey among breast, prostate, and colorectal cancer survivors living in Nova Scotia showed less than half of survivors were meeting PA guidelines [11]. Therefore, interventions focusing on behavior change are necessary to help increase PA levels among these cancer survivors.

An essential step in promoting behavior change is the use of targeted messages to increase motivation for the specific behavior. Investigating the PA correlates and preferences of cancer survivors is important when developing these targeted messages. To date, theory-based behavior change interventions designed to increase PA levels among cancer survivors have employed face-to-face, telephone, email, and print-based methods [9,12-17]. Encouraging results suggest a positive influence of these interventions on PA among cancer survivors.

Recent meta-analyses and reviews [18-25] have summarized the effectiveness of technology when delivering interventions among the general population as well as various chronic disease populations. Overall, the research has found various forms of technology to be effective in facilitating PA behavior. Davies et al [19] reviewed computer-tailored or Web-delivered behavior change interventions across various groups and found effect sizes for change in PA were small but significant in the healthy population (*d*=0.11) in those with chronic disease (*d*=0.19) and in those who were overweight (*d*=0.28). The benefits of using an Internet-delivered program is the efficiency and reach that it can provide.

Face-to-face counseling is time consuming, resource intensive, and requires participants to live near a physical location [18,21,23,24]. This is particularly important in regions with a large rural population such as Nova Scotia, which is approximately 43% rural [26]. The Internet provides people who may not be able to access standard education sessions with an alternative. Having components of the educational content given in oncologist consultations delivered via the Internet could relieve some of the burden on oncologists to deliver the message and help patients to retain information. Previous research into the PA counseling and programming preferences of cancer survivors in Nova Scotia [27] revealed that 76% of the sample had access to the Internet. Approximately 50% of the sample indicated that they would be willing to receive PA information online and would be able to complete the questionnaires online as well.

Currently, there is only one study that examined PA behavior change among cancer survivors using an online delivery [28]. Lee and colleagues [28] randomized 59 women in Seoul, South Korea who completed breast cancer treatment, into either a Web-based self-management PA and diet intervention group developed using the transtheoretical model (TTM) [29] or a control group, which received an educational booklet on PA and diet. They found that the Web-based intervention group increased the proportion of people meeting moderate-intensity activity guidelines of ≥150 minutes more so than the control group (from 33% to 66% versus 35% to 36%, respectively). However, the small sample of nonrepresentative (younger, more educated) breast cancer survivors makes generalizing these results difficult. In addition and similar to many studies using the TTM as a template, this study did not fully operationalize the multidimensional model which is a limitation when determining effectiveness [30].

The primary purpose of this study is to test the feasibility of an Internet-delivered PA behavior change intervention among breast, prostate, and colorectal cancer survivors living in Nova Scotia. A secondary purpose is to examine the preliminary efficacy of the intervention for improving PA and QOL. We hypothesized that it would feasible to use an Internet-delivered program to deliver a behavior change program to breast, prostate, and colorectal cancer survivors living in Nova Scotia. In addition, we hypothesized that the website program would result in an increase in self-reported PA and QOL, although we did not anticipate a statistically significant difference given the feasibility nature of the study.

## Methods

### Study Procedures and Population

Participants were recruited from a sample (N=415) of breast, prostate, and colorectal cancer survivors living in Nova Scotia who had previously taken part in a survey study and had indicated an interest in future studies [11]. The sample was contacted via email, mail, or telephone with an invitation to participate that included an information sheet from the investigators explaining the purpose of the study and instructions on how to proceed if interested, a consent form, and a copy of the primary publication from the previous survey. Eligibility criteria were (1) being able to speak and read English, (2) having access to the Internet, and (3) being able and interested in an Internet-delivered program designed to increase weekly PA levels.

### Design

This study was a pilot 2-group randomized controlled trial to compare a usual care group (no intervention) with an Internet-delivered behavior change group. The focus of the behavior change program specifically was to increase PA in the form of steps or minutes. Eligible participants provided informed consent and completed a baseline questionnaire to gather demographic, behavioral, and PA information prior to randomization.

### Randomization

A rolling blocked randomization was completed after baseline measures were collected to ensure participants did not have an overly long wait to start the intervention. Participants were randomly allocated to one of two groups using a computer generated random numbers list. The 2 groups were the control group—also called usual care (UC)—and the intervention group (UCAN), which consisted of membership in a private online community called *Active Nova Scotia* housed on the PA tracking website UWALK [31] and modified for cancer survivors. Group assignments were generated by a research assistant and assigned after blocks of baseline measures were received to eliminate bias in group allocation. Participants were then notified of their group assignment via email.

### Intervention

Those randomized into the UCAN group were given access to a 9-module behavior change program which was developed using previous print materials as a template [13,32]. The modules were published sequentially on the site as the intervention progressed to increase retention. Information module topics were developed from survey results of the same group [11,27] and were as follows: (1) welcome, general information about the site, types of exercise, and how to gauge intensity; (2) exercise myths, dispelling common exercise myths; (3) exercise safety, tips on how to exercise smart and safe; (4) goals and planning, how to plan and make SMART goals; (5) exercise benefits, specific benefits of exercise for cancer survivors; (6) make it fun, tips on how to keep exercise fun; (7) exercise barriers, tips on how to overcome the most common barriers identified; (8) support network, how others can help you exercise; and (9) relapse, strategies on how to avoid and deal with relapse. Each module remained available to review after the week was concluded. In addition, each module included a video relevant to the current topic featuring the first author to foster a connection and simulate face-to-face interactions.

Aside from the behavior change program, the UCAN group was able to use the UWALK website to track their PA in steps, moderate or vigorous minutes, and flights of stairs. Participants were able to see the progress of other group members as well as their own progress over time. Participants in the UCAN group also received weekly email updates informing them of new information posts as well as a brief summary of their previous weeks PA levels. Emails were developed to offer encouragement to those who were not meeting the guidelines and congratulate those who were sufficiently active. Upon being informed of their group assignment, the UC group was asked to keep their regular exercise routine over the intervention period and they would receive access to the website and the behavior change program once the follow-up questionnaire was completed.

### Feasibility and Efficacy Measures

#### Demographic and Medical Information

All questionnaires were completed online using FluidSurveys (Ottawa, Ontario) software. Information on demographic and medical data was collected through self-report measures and included age, sex, marital status, education level, income, employment status, ethnicity, and height and weight to compute body mass index (BMI). Medical variables included date of diagnosis, cancer site, disease stage, previous treatments, current treatment status, cancer recurrence, and current disease status. Measures for the primary and secondary end points were examined at baseline (preintervention) and at 10 weeks (postintervention).

#### Website Engagement and Usage

Mixpanel analytics were used to track Web-usage statistics to address our primary objective. This tracking program provides information on number of logins, page views, and activity logged. Mixpanel analytics is a measurement tool that shows the effectiveness of a Web page in achieving a goal. It is an easy way to see how visitors use the site and identify which pages are performing well and which are performing poorly. The program tracks “actions” on pages to allow you to identify how a page is being used. They offer a variety of measurement tools to help you learn about your participants including (1) engagement (measures the actions that people take in the website); (2) retention (finds out if people come back); (3) funnel analysis (pinpoints where and why participants are lost); (4) notifications (gets participants to come back with email or push notifications); and (5) people analytics (explores who your participants are and what they do).

#### Program Evaluation and Adherence

To assess program satisfaction, a primary objective, participants randomized to the UCAN group were asked to complete a section examining overall website satisfaction and usefulness of the different program features. The questions were adapted from a recent Web-based PA intervention for people with type 2 diabetes [22], which was in turn developed from the Health-*e*Steps [33] and Diabetes NetPLAY programs [34]. The items used a 4-point Likert-type scale ranging from “strongly disagree” to “strongly agree” for the following statements: “I enjoyed the Active Nova Scotia program,” “If I had any concerns I knew who to contact,” “I would continue to participate in the Active Nova Scotia program,” “I increased my PA because I was in this study,” “This study made me more aware of the amount of PA I get each day,” “The topics for each information post were useful and relevant,” “I liked the videos for the information posts,” “The videos in the information posts were not burdensome on my computer,” I was able to easily find my way around the website,” “I was able to easily record my PA on the website,” “I would recommend this website to other people,” and “I will continue to use the website now that the Active Nova Scotia program has finished.” These 12 items were supplemented by 4 open-ended questions to indicate likes, dislikes, and recommendations for future development.

#### Physical Activity Behavior

To address our secondary objective, PA was measured using a modified version of the validated Leisure Score Index (LSI) from Godin’s Leisure Time Exercise Questionnaire (LTEQ) [35]. Participants were asked to recall the average frequency and duration of any vigorous (heart beats rapidly, sweating), moderate (not exhausting, light perspiration), and light (minimal effort, no perspiration) intensity aerobic PA, as well as resistance exercise (lifting weights, sit-ups, pushups, therabands) in a typical week over the past month. PA sessions had to be at least 10 minutes long and performed during their free time and not occupational. The percentage of participants meeting PA guidelines was calculated using the 2008 PA Guidelines for Americans [36], which have been recommended for cancer survivors by the American College of Sports Medicine [37] and the American Cancer Society [3]. The guidelines indicate that cancer survivors should perform either 75 minutes of vigorous activity a week, 150 minutes of moderate activity a week, or a combination that double weights the vigorous minutes. PA minutes were calculated as moderate minutes plus two times vigorous minutes and then transformed into 2 categories (1) not meeting guidelines (≤149 minutes) or (2) meeting guidelines (≥150 PA minutes). The percentage of participants meeting strength guidelines was defined as those engaging in two or more sessions of strength exercise per week. Strength minutes were calculated by multiplying the average minutes per session by strength frequency. Total exercise minutes were calculated by adding PA minutes and strength minutes.

#### Quality of Life

As part of the secondary objective, QOL was assessed by the validated Functional Assessment of Cancer Therapy-Fatigue (FACT-F) scale which includes the 27 items from the FACT-General (FACT-G) scale plus the 13-item fatigue subscale [38,39]. The FACT-G consists of physical well-being, functional well-being, emotional well-being, and social well-being. On all scales, higher scores indicate better QOL. QOL was also assessed using the Medical Outcomes Study 36-Item Short Form (SF-36) [40], which contains 36 items that produce 8 health domains with multi-item scales. *Physical functioning* evaluates limitations in physical activities, such as walking and climbing stairs. *Role limitations* as a result of *physical* or *emotional* health conditions measure problems with work or other daily activities. *Bodily pain* assesses limitations caused by pain, and *vitality* measures levels of energy and tiredness. *Social functioning* examines the effect of physical or emotional health on normal social activities, and *mental health* evaluates happiness, nervousness, and depression. The *general health perceptions* questions examine personal health and the expectation of changes in health. A single item assesses *change in perceived health* during the last year. All items used a Likert-type scale of varying points.

### Statistical Analysis

All analyses were performed using PASW Statistics 22 (PASW Inc., Chicago, IL, USA). Feasibility was assessed using recruitment rate, website satisfaction, and usage statistics gathered from UWALK and Mixpanel. Chi-square and analyses of variance (ANOVAs) were performed to determine the differences between the intervention groups for PA behavior and QOL. Analyses of covariance (ANCOVAs) were also conducted to adjust for baseline value when comparing intervention groups. Results were interpreted for statistical trends as well as for potential clinical significance. Using a two-tailed alpha of *P*≤.05, the study had 80% power to detect medium standardized effects (*d*=0.50) after adjustment for covariates with 45 participants per condition. Trends were defined as *P*<.10 and potential clinical significance as a standardized effect size of *d*≥0.33 [41]. Intention-to-treat protocol was adhered to for all analyses. Responders and nonresponders were compared to determine any differences. Based on the higher than expected number of participants meeting PA guidelines at baseline, subgroup analyses were conducted for those with less than 150 minutes versus 150 minutes or more of total exercise.

## Results

**Figure 1:**
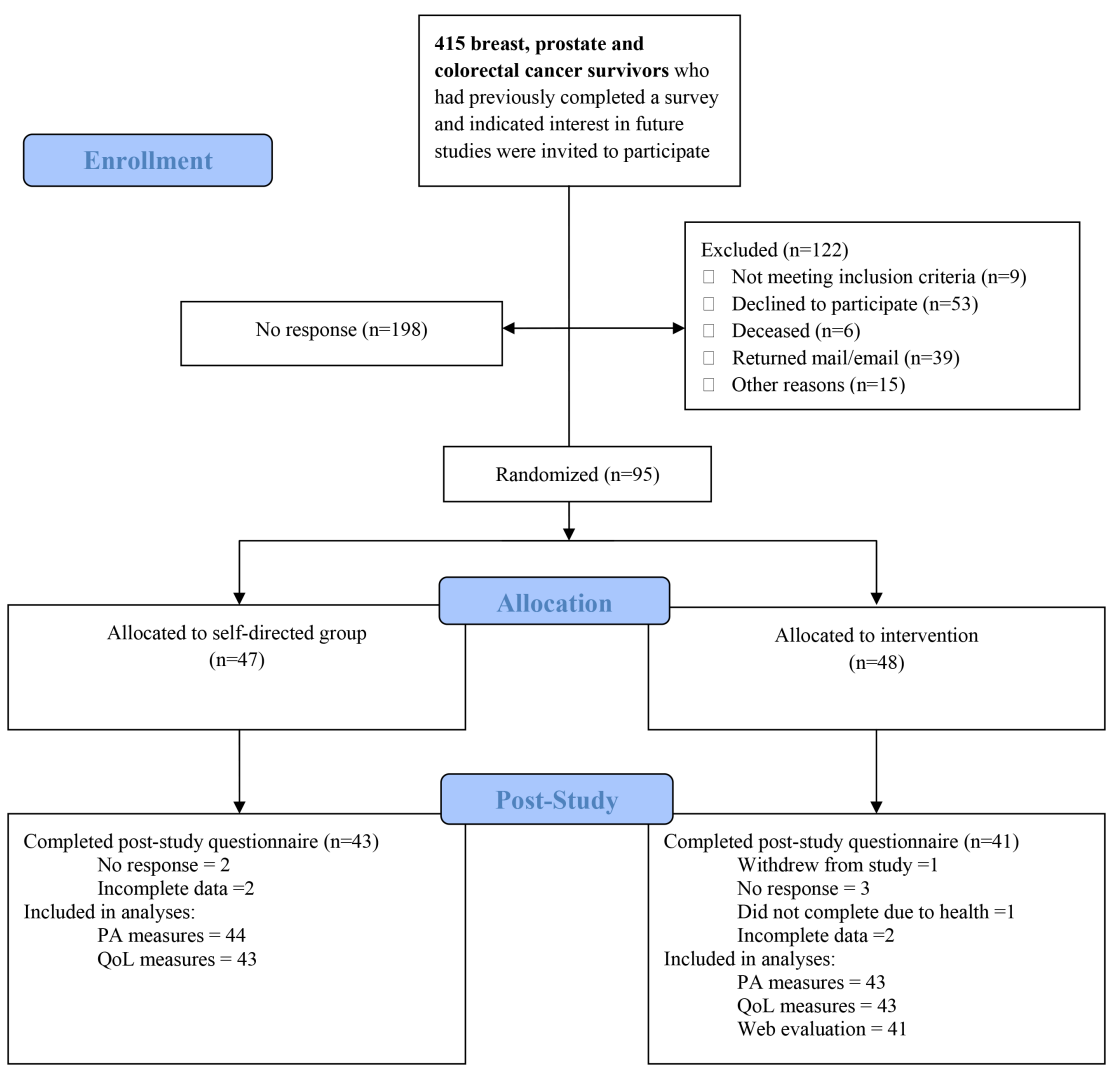
Detailed flow of study participants from invitation through postintervention.

The detailed flow of participants from invitation to randomization can be found in Figure 1. Of the 415 cancer survivors contacted, 197 (47.5%, 197/415) did not respond and 98 (23.6%, 98/415) were excluded for various reasons. Of the 120 (28.9%, 120/415) survivors who expressed interest, 25 were excluded for not meeting inclusion criteria where 9 (36%, 9/25) did not have Internet access or a computer, 4 (16% (4/25) did not reply after initial interest, and 12 (48%, 12/25) contacted us after recruitment had closed. Of the 95 cancer survivors, 48 (50%, 48/95) were randomized into the UCAN group and 47 (50%, 47/95) into the UC group, resulting in a 22.9% (95/415) recruitment rate. During the study 1 person withdrew due to personal issues. At the postintervention evaluation, 84 (88%, 84/95) completed 100% of the poststudy survey. Among those who did not fully complete the survey, 5 (45%, 5/11) were nonresponders, 5 (45%, 5/11) had incomplete data, and 1 (9%, 1/11) had non-cancer-related health issues. At baseline, the majority of the sample was female (56%, 53/95), married (86%, 82/95), more educated (77%, 73/95), had higher income (50%, 47/95), breast cancer (51%, 48/95), over 5 years since diagnosis (85%, 81/95), currently disease free (96%,91/95), and indicated a perceived general health of good or better (95%, 90/95). Mean age and BMI were 65.1 years and 27.6 kg/m^2^, respectively. The majority of participants were not meeting minimum PA guidelines (54%, 51/95). Detailed demographic and medical information can be found in Table 1.

**Table 1:** Demographic, medical, and behavioral characteristics of cancer survivors in Nova Scotia, Canada, from September to October 2014.

Demographic/behavior variables		Overall (N=95) n (%)	UC (N=47) n (%)	UCAN (N=48) n (%)
**Gender**				
	Female	53 (56)	26 (55)	27 (56)
Age, mean (SD)		65.1 (8.5)	65.7 (8.6)	64.5 (8.4)
**Ethnic origin**				
	White	94 (99)	46 (98)	48 (100)
**Marital status**				
	Married	82 (86)	41 (87)	41 (85)
**Education**				
	Postsecondary	73 (77)	41 (87)	32 (67)
**Family income**				
	< 60,000	32 (34)	18 (38)	14 (30)
	≥ 60,000	47 (50)	22 (47)	25 (52)
	Prefer not to answer	16 (17)	7 (15)	9 (19)
**Employment**				
	Not employed	66 (69)	34 (72)	32 (67)
**Smoking status**				
	Never	43 (45)	23 (49)	20 (42)
	Ex-smoker	47 (50)	19 (40)	28 (58)
	Current smoker	5 (5)	5 (11)	0 (0)
**Alcohol consumption**				
	Never drink	21 (22)	8 (17)	13 (27)
	Social	60 (63)	30 (64)	30 (63)
	Regular	14 (15)	9 (19)	5 (10)
**Meeting PA guidelines**				
	No	51 (54)	25 (53)	26 (54)
**Dog owner**				
	Yes	21 (22)	11 (23)	10 (21)
**Cancer type**				
	Breast	48 (51)	23 (49)	25 (52)
	Prostate	27 (28)	14 (30)	13 (27)
	Colorectal	20 (21)	10 (21)	10 (21)
**Disease stage**				
	Localized	83 (88)	42 (90)	41 (86)
	Metastasized	6 (6)	2 (4)	4 (8)
	Don’t know	6 (6)	3 (6)	3 (6)
**Surgery**				
	Yes	90 (95)	46 (98)	44 (92)
**Radiation therapy**				
	Yes	43 (45)	22 (47)	21 (44)
**Chemotherapy**				
	Yes	41 (43)	16 (34)	25 (52)
**Hormone therapy**				
	Yes	25 (26)	10 (21)	15 (31)
**Current treatment status**				
	No treatment	75 (79)	40 (85)	35 (73)
**Recurrence**				
	Yes	6 (6)	0 (0)	6 (12)
**Current disease status**				
	Disease free	91 (96)	47 (100)	44 (92)
Time (years) since diagnosis, mean (SD)		6.6 (2.6)	6.4 (2.9)	6.8 (2.4)
**General health**				
	Very good/excellent	48 (51)	28 (60)	42 (42)
	Good	42 (44)	18 (38)	24 (50)
	Poor/Fair	5 (5)	1 (2)	4 (8)
**Comorbidity status**				
	No comorbidities	15 (16)	5 (11)	10 (21)
	1-2 comorbidities	52 (55)	25 (53)	27 (56)
	≥3 comorbidities	28 (29)	17 (36)	11 (23)
**BMI (kg/m** ^**2**^ **), mean (SD)**		27.6 (4.4)	27.1 (3.9)	28.1 (4.9)
	Healthy weight	32 (34)	16 (34)	16 (33)
	Overweight	33 (35)	20 (43)	13 (27)
	Obese	30 (31)	11 (23)	19 (40)

Based on data from our original survey, we were able to compare study participants (n=95) to the nonparticipants (n=320). We found that study participants were more likely to be meeting PA guidelines (*P*=.005), have breast cancer (*P*=.002), previous hormone therapy (*P*=.013), be married (*P*=.024), more educated (*P*=.014), have higher income (*P*<.001), be employed (*P*=.044), have a stronger preference for receiving PA information via the Internet (*P*=.002) or email (*P*<.001), and a weaker preference for receiving information face-to-face (*P*=.019).

### Website Usage

**Figure 2:**
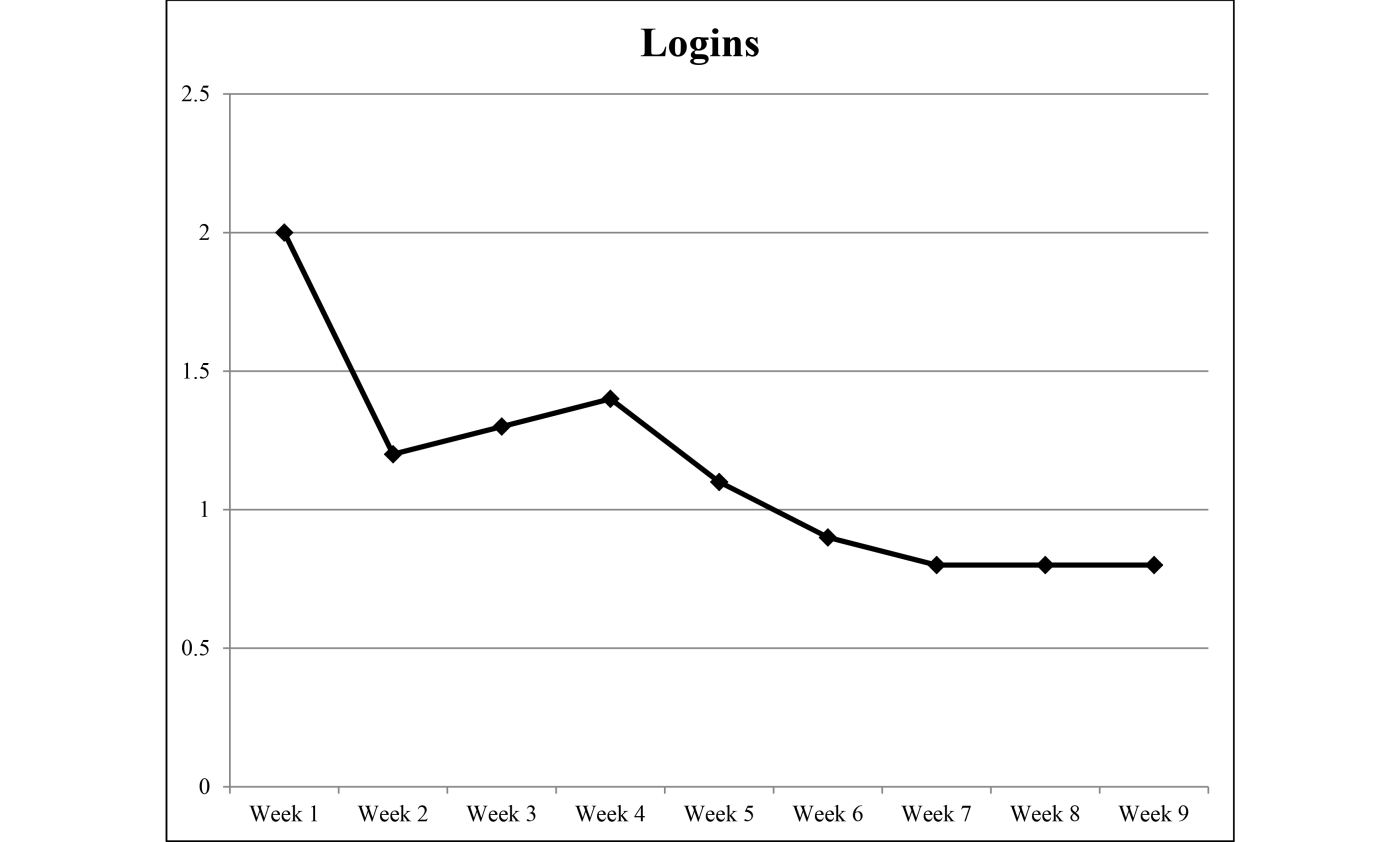
Average number of logins per week during the 9-week study period from September to December 2014.

**Figure 3:**
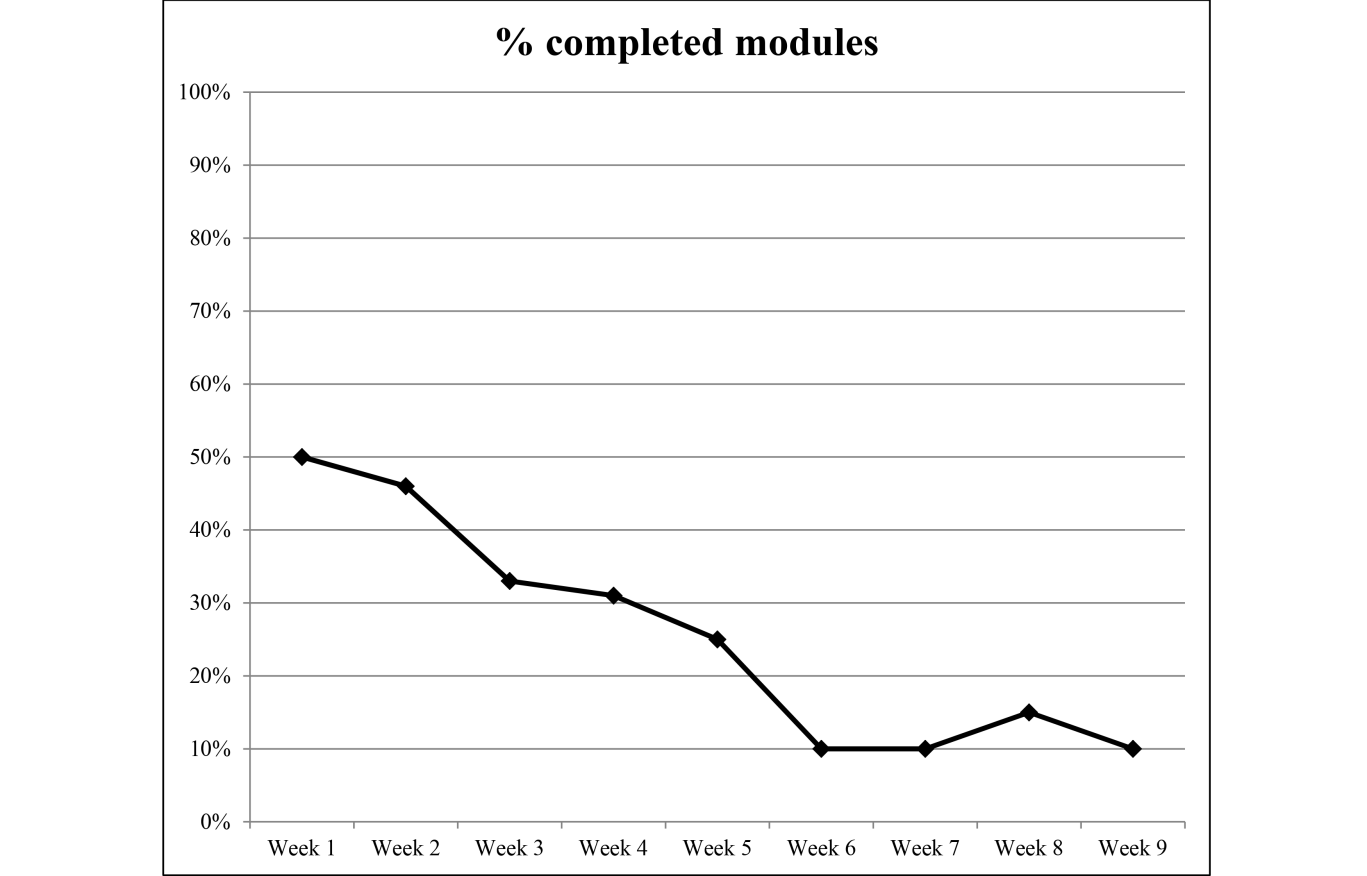
Percentage of completed modules per week during the 9-week study period from September to December 2014.

**Figure 4:**
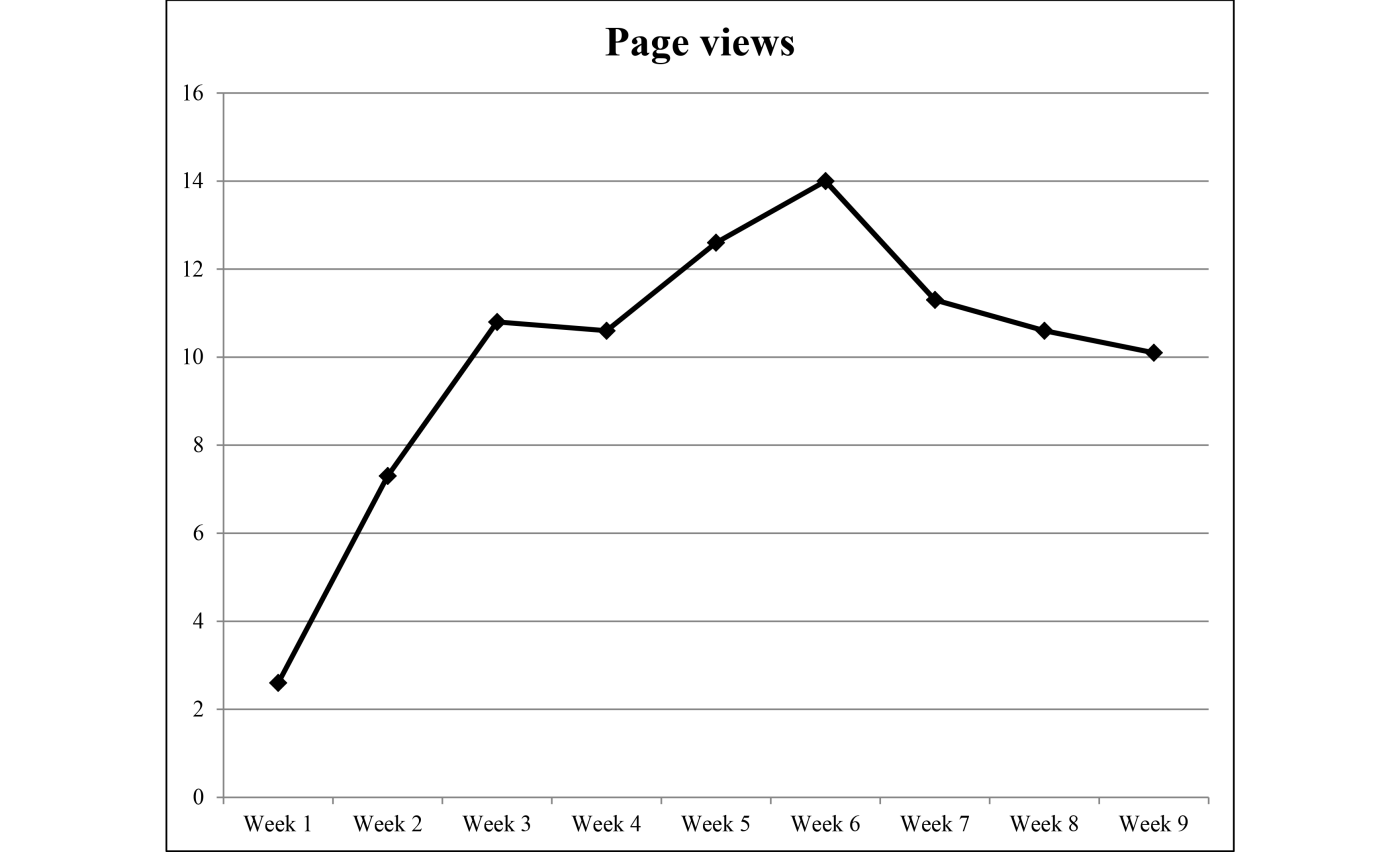
Average number of page views per week during the 9-week study period from September to December 2014.

**Figure 5:**
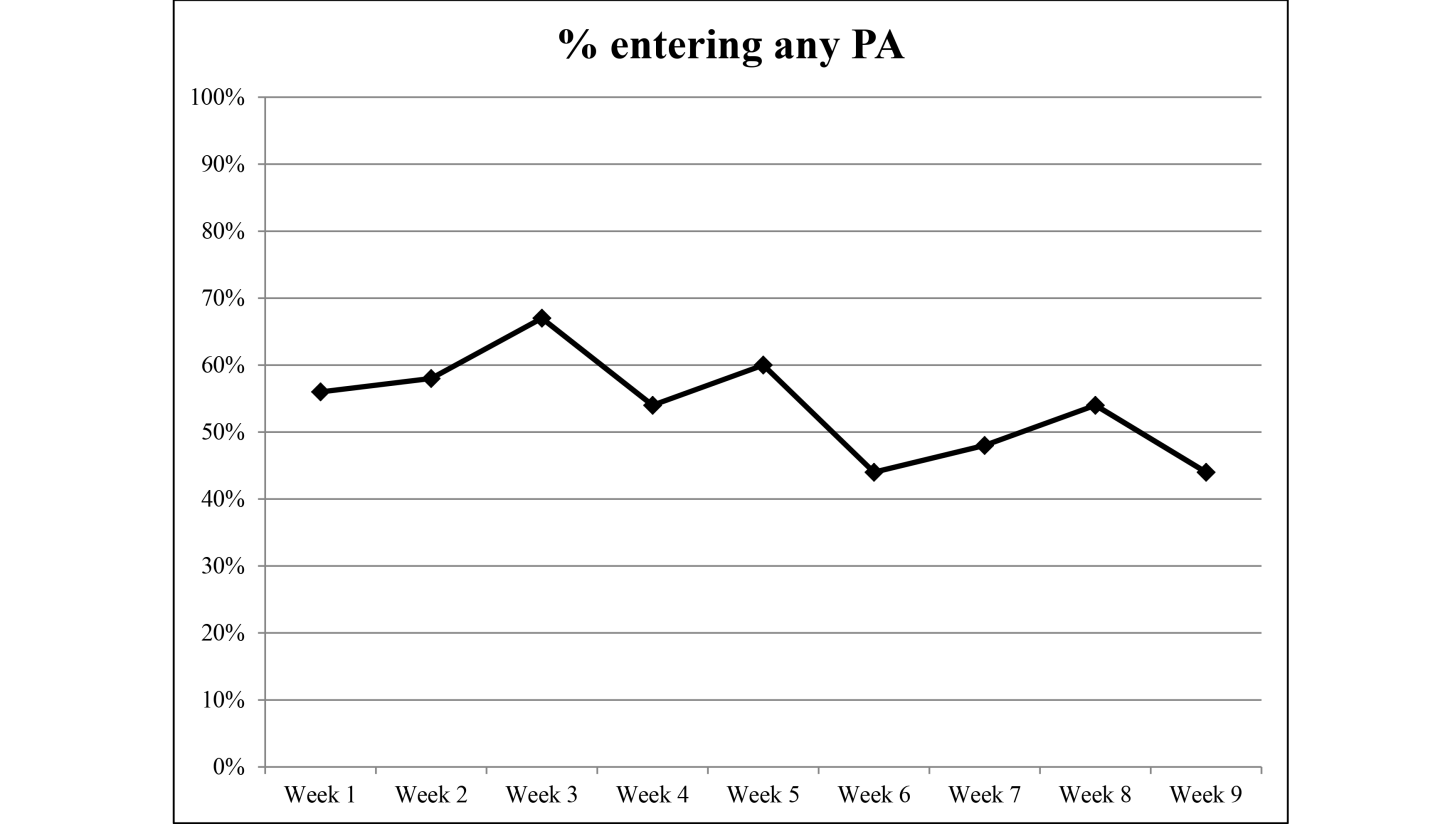
Percentage of participants entering PA per week during the 9-week study period from September to December 2014.

Detailed weekly Web statistics are shown in Figures 2-5. The overall average number of logins was 10.3 for the 9-week duration of the intervention. There were 2293 individual PA events logged over 1085 days (average 23 days per participant) and 4319 page views recorded. The most frequently visited page was the log page where participants entered their PA data. The modules were visited 213 times over the length of the study with an overall read rate of 26%. Moreover, 94% (45/48) of participants logged in at least once, 85% (41/48) recorded PA at least once, and 67% (32/48) viewed the modules at least once.

### Intervention Satisfaction

With regard to the intervention program, 73% (30/41) said they enjoyed the Active Nova Scotia program, 63% (26/41) would be willing to continue participating, 46% (19/41) indicated they increased their PA because of this program, 71% (29/41) said they were more aware of the amount of PA they get each day, and 73% (30/41) thought the information in the weekly modules was useful and relevant. About half of the participants (51%, 21/41) liked the video posts and felt they were not too burdensome on their computer. When evaluating the website, 68% (28/41) were able to easily navigate and enter PA information on the site. When asked if they would recommend the site to others, 64% (26/41) indicated yes and 39% (16/41) said they would continue using the site after the study had finished.

### Effect on Physical Activity Behavior

**Figure 6:**
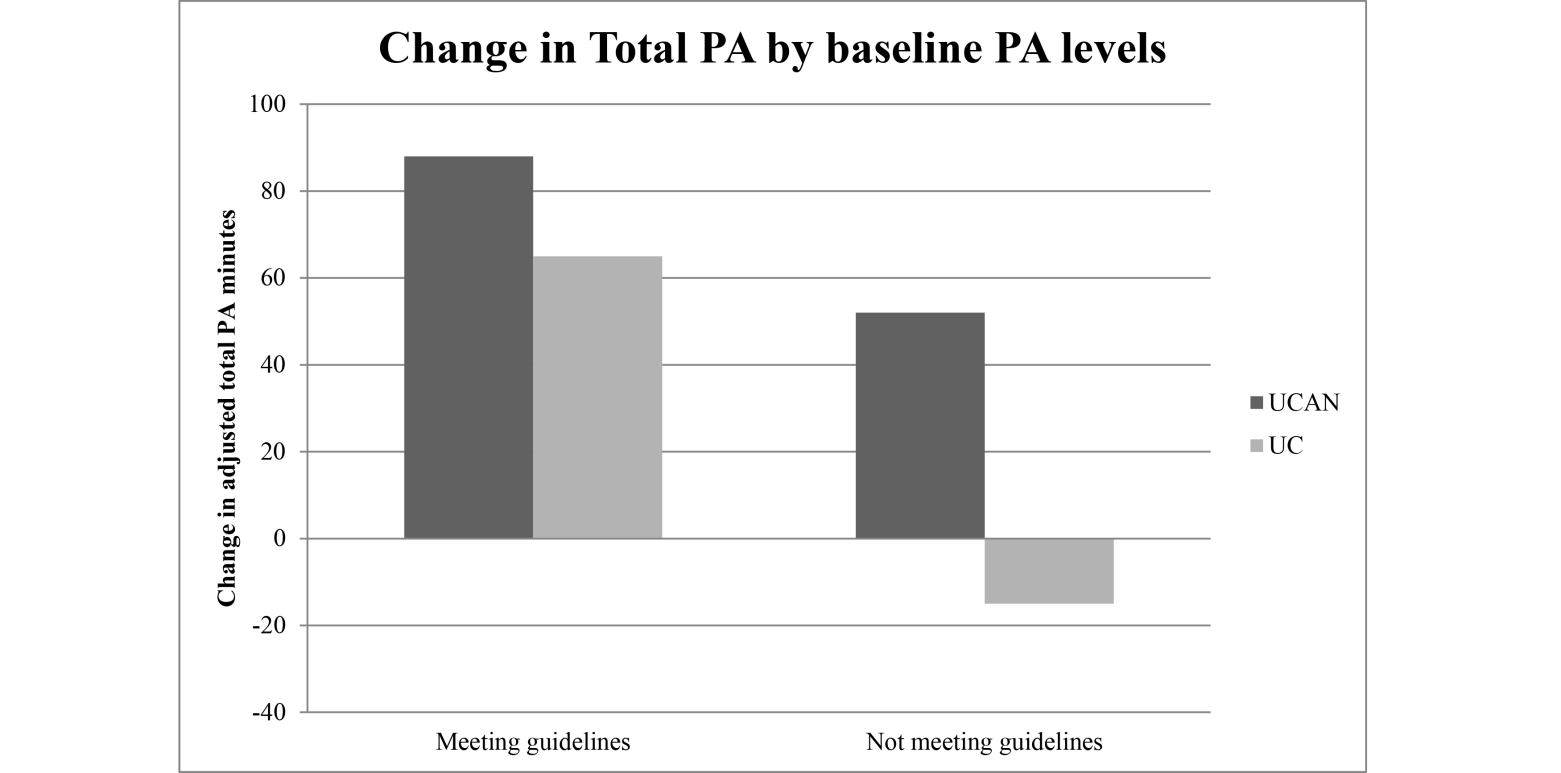
Change in adjusted total PA by meeting guidelines at baseline. This figure shows the trend in PA minutes between the UCAN and UC group when analyzing the subgroups for those meeting PA guidelines at baseline versus those not meeting guidelines at baseline.

The differences in PA behavior between the UC and UCAN groups at baseline and postintervention are described in Table 2. Overall, the adjusted between-group mean change scores favored the UCAN group; however, there were no significant differences between the groups in any PA measure. The adjusted between-group difference for total exercise minutes was 42 (95% CI -65 to 150; *P*=.44, *d*=0.17) in favor of UCAN. The adjusted between-group difference for strength training frequency achieved a meaningful difference of 0.5 (95% CI -0.2 to 1.1; *P*=.14, *d*=0.34). The subgroup analysis of the changes in total exercise minutes by baseline PA levels is illustrated in Figure 6. Among those not meeting guidelines at baseline (54%, 51/95), the UCAN group (54%, 26/48) increased their PA levels by 52 minutes (95% CI -74 to 178) while the UC group (53%, 25/47) decreased by 15 minutes (95% CI -140 to 109); whereas among those meeting guidelines, the UCAN (46%, 22/48) and UC group (47%, 22/47) increased PA by 88 (95% CI -55 to 230) and 65 minutes (95% CI -57 to 186), respectively.

**Table 2:** Effects of Internet-delivered behavior change PA program on PA in Nova Scotian cancer survivors from September to December 2014 (N=87).

Outcome (N=87)		Baseline, mean (SD)	Poststudy, mean (SD)	Mean change, mean (95% CI)	Adjusted between group difference in mean change^a^, mean (95% CI); *P*, *d*
**Total exercise minutes** ^b^					
	UC	212 (216)	241 (197)	30 (-18 to 77)	42 (-65 to 150); .44, 0.17
	UCAN	231 (269)	294 (354)	64 (-45 to 172)	
**Total aerobic minutes** ^c^					
	UC	194 (207)	222 (183)	29 (-19 to 76)	29 (-65 to 123); .55, 0.04
	UCAN	208 (253)	258 (302)	50 (-47 to 147)	
**Moderate aerobic minutes** ^d^					
	UC	117 (140)	128 (110)	11 (-32 to 53)	14 (-36 to 63); .58, 0.12
	UCAN	112 (132)	140 (132)	27 (-22 to 77)	
**Vigorous aerobic minutes** ^d^					
	UC	39 (66)	47 (71)	9 (-7 to 25)	6 (-27 to 38); .73, -0.03
	UCAN	48 (91)	59 (109)	11 (-20 to 42)	
**Meeting aerobic guidelines**					
	UC	50% (51%)	68% (47%)	19% (3-34)	-9% (-27 to 10%); .36, -0.23
	UCAN	47% (51%)	58% (49%)	12% (-5 to 28)	
**Strength frequency**					
	UC	0.7 (1.2)	0.8 (1.3)	0.1 (-0.2 to 0.4)	0.5 (-0.2 to 1.1); .14, 0.34
	UCAN	0.9 (1.5)	1.4 (2.2)	0.5 (-0.02 to 1.0)	
**Strength minutes**					
	UC	18 (35)	19 (36)	2 (-6 to 9)	12 (-10 to 35); .28, 0.04
	UCAN	23 (45)	36 (84)	14 (-8 to 36)	
**Meeting strength guidelines** ^e^					
	UC	25% (44%)	27% (45%)	2% (-12 to 17)	6% (-11 to 23%); .48, 0.18
	UCAN	28% (45%)	35% (48%)	7% (-5 to 19)	

^a^Difference in mean change adjusted for baseline value.

^b^Total exercise minutes was computed by adding total aerobic minutes to total strength minutes.

^c^Total aerobic minutes was computed using moderate minutes plus 2 times the vigorous minutes.

^d^Capped at 420 minutes per week.

^e^Strength guidelines is engaging in strength exercise ≥2 times per week.

### Quality of Life

The general and cancer-specific QOL measures at baseline and postintervention are summarized in Tables 3 and 4. Change in the SF-36 measure of mental health favored the UC group with a mean change score of -2.9 (95% CI -5.1 to -0.6; *P*=.014, *d*=0.37). All other measures were nonsignificant. No measures met the point difference that indicates clinical significance (SF-36=3 point difference; FACT subscales=2-3 point difference).

## Discussion

### Principal Findings

Our study is one of the first to use an online platform to deliver a theory-based PA behavior change program to cancer survivors, and the first to target Nova Scotian cancer survivors. Based on recruitment, retention, and participant evaluation of the program we believe that the program is feasible. In addition, there were trends suggesting the potential effectiveness of the program for promoting PA, especially in cancer survivors who were inactive at baseline. Engagement in the program and influencing QOL, however, remain a challenge for distance-based program delivery.

Our expression of interest rate (29%, 120/415) and recruitment rate (23%, 95/415) was similar to other studies [22,28]. The previous study among cancer survivors resulted in a 17% recruitment rate but used community- and clinical-based recruitment methods that were unable to track the initial reach of the invitation [28]. Our postintervention retention (88%, 84/95) was higher than the majority of previous studies using the Internet as a delivery method [19,28,42]. Large attrition is common among Internet-based interventions [18,20-22,42] and like previous research we had slightly higher attrition in the intervention group (15% vs 9%) despite the high satisfaction ratings [22]. It is difficult to pinpoint the reason for such high dropout rates in Web-based studies but previous research indicates it is easier for participants to disengage from Web-based interventions [43]. Using strategies to increase the contact between user-to-user and user-to-researcher may help increase the connection and make the intervention meaningful to the participant [23].

Engagement in our study was fairly low compared to other Internet-based studies [22,28]. The modules had a completion rate of 26% (111/432 potential completions). As with logins, the number of completed modules dropped after the first few weeks. Our average number of logins was 10.3 per person. This equals about once per week per person which may be insufficient to induce PA behavior change. This is similar to other studies using Internet delivery [24,42]. A meta-analysis by Davies et al [19] found the average number of logins per-person-per-week was 3.08 across 11 studies. One potential reason for our lower login average is that the website was able to automatically pull data from devices such as the FitBit without the participants having to login. One recent suggestion for increasing user engagement is to allow user-generated content (eg, creating a post to add to the newsfeed) [44] which may increase user “buy-in.” This method, however, requires close monitoring as information would need to be vetted to ensure accuracy and relevance. Retaining and engaging participants remain an issue among Internet-delivered behavior change programs.

**Table 3:** Effects of Internet-delivered PA program on generic QOL in Nova Scotian cancer survivors from September to December 2014 (N=86).

Outcome		Baseline, mean (SD)	Poststudy, mean (SD)	Mean change, mean (95% CI)	Adjusted between group difference in mean change^a^, mean (95% CI); *P*, *d*
**Physical functioning**					
	UC	49.1 (9.7)	50.1 (7.0)	1.0 (-1.5 to 3.5)	-0.6 (-3.3 to 2.2); .68, 0.18
	UCAN	47.8 (7.9)	49.0 (8.0)	1.1 (-1.3 to 3.5)	
**Role physical**					
	UC	50.4 (7.5)	49.4 (8.3)	-0.9 (-3.4 to 1.6)	-1.0 (-4.3 to 2.2); .53, -0.06
	UCAN	48.5 (8.6)	47.0 (11.0)	-1.5 (-3.8 to 0.7)	
**Bodily pain**					
	UC	51.0 (8.4)	51.5 (9.1)	0.5 (-1.9 to 2.9)	-1.6 (-4.8 to 1.5); .30, -0.23
	UCAN	49.0 (7.6)	48.6 (9.0)	-0.5 (-2.8 to 1.9)	
**General health**					
	UC	46.0 (5.9)	47.4 (6.2)	1.4 (-0.4 to 3.2)	-1.8 (-4.2 to 0.5); .12, -0.27
	UCAN	46.7 (6.4)	46.1 (7.6)	-0.6 (-2.3 to 1.1)	
**Vitality**					
	UC	44.9 (7.9)	45.2 (8.3)	0.3 (-1.2 to 1.8)	-1.4 (-3.9 to 1.0); .25, -0.09
	UCAN	45.7 (7.2)	44.5 (9.3)	-1.2 (-3.2 to 0.7)	
**Social functioning**					
	UC	51.6 (8.7)	51.0 (8.8)	-0.6 (-2.9 to 1.7)	-1.7 (-4.9 to 1.5); .30, -0.00
	UCAN	50.3 (8.4)	48.4 (10.3)	-1.9 (-4.4 to 0.6)	
**Role emotional**					
	UC	51.8 (7.1)	51.1 (8.3)	-0.7 (-3.8 to 2.4)	1.5 (-5.3 to 2.4); .44, 0.00
	UCAN	50.6 (8.0)	49.1 (10.5)	-1.5 (-4.5 to 1.5)	
**Mental health**					
	UC	44.7 (4.8)	44.9 (5.8)	0.3 (-0.9 to 1.4)	-2.9 (-5.1 to -0.6); .014, -0.37
	UCAN	45.0 (5.6)	42.3 (8.5)	-2.6 (-4.6 to -0.6)	
**Physical health component**					
	UC	49.7 (7.8)	50.4 (7.5)	0.7 (-1.3 to 2.8)	-0.8 (-3.3 to 1.8); .55, -0.09
	UCAN	48.3 (8.0)	48.8 (7.9)	0.5 (-1.4 to 2.4)	
**Mental health component**					
	UC	47.6 (6.0)	47.1 (7.3)	-0.5 (-2.4 to 1.3)	-2.2 (-5.2 to 0.8); .14, -0.10
	UCAN	47.7 (7.6)	45.0 (10.2)	-2.7 (-5.3 to -0.2)	

^a^Difference in mean change adjusted for baseline value.

**Table 4:** Effects of Internet-delivered PA program on cancer-specific QOL in Nova Scotian cancer survivors from September to December 2014 (N=86).

Outcome		Baseline, mean (SD)	Poststudy, mean (SD)	Mean change, mean (95% CI)	Adjusted between group difference in mean change^a^, mean (95% CI); *P*, *d*
**Physical well-being**					
	UC	24.4 (4.0)	24.4 (3.7)	-0.1 (-0.9 to 0.8)	-0.6 (-1.8 to 0.5); .28, -0.06
	UCAN	25.1 (2.5)	24.2 (3.7)	-0.8 (-1.7 to 0.04)	
**Social well-being**					
	UC	19.8 (5.9)	19.3 (5.9)	-0.6 (-1.8 to 0.7)	0.5 (-1.2 to 2.1); .57, 0.20
	UCAN	21.2 (5.5)	20.8 (5.6)	-0.4 (-1.7 to 0.8)	
**Emotional well-being**					
	UC	20.6 (3.6)	20.3 (4.5)	-0.3 (-1.8 to 1.1)	0.3 (-2.0 to 1.3); .69, 0.22
	UCAN	20.2 (3.6)	19.8 (3.7)	-0.4 (-1.7 to 0.9)	
**Functional well-being**					
	UC	23.3 (4.0)	22.8 (5.5)	-0.5 (-2.0 to 1.0)	-0.4 (-2.3 to 1.4); .64, -0.11
	UCAN	23.1 (4.3)	22.2 (5.1)	-0.9 (-2.0 to 0.2)	
**Fatigue symptoms**					
	UC	41.1 (11.9)	38.2 (8.2)	-2.9 (-5.1 to -0.7)	0.2 (-2.2 to 1.8); .85, 0.06
	UCAN	41.7 (8.5)	38.4 (6.4)	-3.4 (-5.2 to -1.6)	
**FACT-G**					
	UC	88.2 (14.1)	86.8 (14.3)	-1.5 (-4.8 to 1.8)	0.9 (-5.2 to 3.5); .69, 0.06
	UCAN	89.6 (11.7)	87.0 (15.0)	-2.6 (-5.6 to 0.5)	
**FACT-F**					
	UC	129.4 (23.7)	125.0 (19.8)	-4.4 (-9.0 to 0.2)	-1.1 (-6.5 to 4.4); .70, 0.04
	UCAN	131.3 (17.6)	125.4 (20.0)	-5.9 (-9.8 to -2.1)	
**TOI-F**					
	UC	88.9 (18.7)	85.5 (15.0)	-3.5 (-7.1 to 0.1)	-1.3 (-5.3 to 2.7); .51, -0.08
	UCAN	89.9 (13.2)	84.8 (13.5)	-5.1 (-8.0 to -2.2)	

^a^Difference in mean change adjusted for baseline value.

Overall, the program was very well received among participants in the UCAN group despite the low usage numbers. This is similar to other Internet-based PA programs [22,28,42,45]. Most participants felt that the information provided was useful and relevant and they indicated that they were more aware of their level of daily activity. They also indicated they liked the weekly posts and videos and would be interested in continuing with the ANS program. Participants evaluated the website favorably and said they would recommend it to a friend but the majority indicated they would not continue using the site with the program finished. Very few participants contacted the study coordinator with issues related to using the website.

Engagement seems to be the biggest hurdle in testing and implementing Internet-delivered interventions. Vandelanotte et al [23] evaluated freely accessible websites that promote PA and found that many did not use tools such as self-monitoring, goal setting, and targeted feedback despite the supporting evidence [21,24,25,46]. An aspect found to be useful that our study lacked is a method of users generating their own content. Despite having a “news feed,” our users were not able to directly message other participants which has been shown to increase effectiveness of Web-based interventions [25]. Standardizing the components of behavior change websites and thoroughly testing them will allow researchers to determine which are most effective among various populations.

As expected based on the small sample size of this pilot study, there were no significant between-group differences in any PA measure, one component we used to determine efficacy. Nevertheless, after adjusting for baseline measures, the UCAN group increased total exercise by 42 minutes more than UC (29 aerobic minutes plus 12 strength minutes) which translated into a small standardized effect size of *d*=0.17. This is slightly higher than the overall effect size of *d*=0.12 found by Davies and colleagues [19]. Moreover, the largest effect of the intervention was for strength training frequency where the UCAN group added a half day per week compared to the UC group (*d*=0.34). Despite the majority of PA measures showing nonsignificant increases favoring the UCAN group, the percentage of participants meeting guidelines, based on the standard cutpoint of 150 minutes, showed a nonsignificant potentially meaningful change favoring the UC group. This finding is somewhat arbitrary because it is dependent on the baseline level of PA (ie, where participants start). There were more UC participants in the “insufficiently active” category than UCAN at baseline; consequently, the smaller increase in minutes per week may have been enough to result in a larger proportion meeting guidelines.

The previous research among cancer survivors [28] and the meta-analysis by Davies and colleagues [19] found that computer-tailored PA programs had positive effects on PA. Previous reviews also indicate that Internet-delivered interventions have positive effects on PA levels [47-49]. One possible explanation for the modest effect of our intervention is the relatively high percentage of participants meeting PA guidelines at baseline (46%, 44/95). Our invitation was to any cancer survivor who wanted to increase his/her PA with the assumption that only less active people would volunteer for such a study. Moreover, we included those meeting the guidelines because research has shown that even more health benefits can be gained by increasing activity levels to 300 or more minutes per week [36,37].

After performing an exploratory subgroup analysis we found a suggestion that the program may be more effective for those who were not meeting guidelines at baseline. Among those not meeting guidelines at baseline, the UCAN group increased their PA levels by 52 minutes while the UC group decreased by 15 minutes (Figure 3). Among those meeting guidelines, the UCAN and UC groups increased PA by 88 and 65 minutes, respectively. The suggestion that PA behavior change programs are most beneficial to those least active is similar to previous research [19]. Targeting specific populations that have lower than average PA levels (ie, cancer survivors, inactive population) may have an even larger effect on clinical and public health outcomes [19].

Not surprisingly, our study did not find any beneficial changes in QOL measures, the second component used to determine efficacy. In fact, the only significant finding was a negative effect on mental health (*P*=.014, *d*=0.37). It is common to find no significant benefits to QOL among distance-based PA interventions for cancer survivors even when PA increases are noted [15,50]. Similar to the PA measures, some studies have found significant improvements in aspects of QOL at postintervention that were not sustained when assessed at follow-up [32,51-57]. Over the course of the study intervention, 14 (29%, 14/48) intervention participants contacted the study coordinator indicating they were having physical or personal issues, which may be a possible explanation for the negative trend in QOL evident in this study. Based on qualitative comments left by participants at the postintervention survey, many felt that the QOL measures used did not apply to them as it had been so long since diagnosis. Approximately 85% (81/95) of the study sample was over 5 years since diagnosis. It may be that the measures used to assess QOL are more applicable to patients on treatments. Despite our inclusion of the generic SF-36, it may be beneficial to include long-term cancer-specific QOL measures for studies among long-term cancer survivors to see if they would be more applicable.

This is the first study to deliver a computer-tailored, Web-delivered PA behavior change intervention to Nova Scotian cancer survivors, and one of the first in any cancer survivor group. This study showed that some cancer survivors are interested and willing to receive PA information through the Internet; however, modifications to the website are necessary to optimize the effectiveness. Limitations of this study are the use of self-report data, selection bias toward those more motivated and Internet savvy, the low usage rate overall, and the decline in usage over the intervention period. Despite the user-friendly website we used to pilot this program, there were comments about confusion on how to use the site and find our information. In the future, we would recommend ensuring a separate site that would be able to house the information in a more prominent position.

Our original study [11] invited people to participate in a PA survey, leading to a selection bias for those motivated to engage in PA. It is common in nonblinded studies to have self-selection bias among participants. Despite this, we were still surprised at the number of participants in the intervention meeting guidelines (46%, 44/95). It may be that the most motivated and active of the previous highly motivated and active survey sample were the ones to come forward for this intervention. In addition, our participants were more likely to prefer receiving information via the Internet which may also bias results. When being asked to participate in an online study, those who prefer this method are more likely to come forward. However, if we were to exclude those already active, our sample size would have been reduced by almost half. Previous preference research found that those who preferred Web-based interventions were more likely to have higher Internet use and higher PA participation [58]. More research into preferences for Internet delivery PA interventions should be explored.

### Conclusions

In conclusion, using a Web-based platform to deliver a PA behavior change intervention to cancer survivors may be a feasible alternative to other methods of information delivery. There was a trend toward increased activity in the UCAN group when compared to the UC group, especially among inactive cancer survivors, although no significant differences were found. User engagement remains a challenge and future research should incorporate as many of the tools previously found to be effective among Web-based interventions to increase engagement and maintain PA behavior.

## Abbreviations

BMI: body mass index
FACT-F: Functional Assessment of Cancer Therapy-Fatigue
FACT-G: Functional Assessment of Cancer Therapy-General
PA: physical activity
QOL: quality of life
SF-36: Medical Outcomes Study 36-Item Short Form
TOI-F: Trial Outcome Index-Fatigue
TTM: transtheoretical model
UC: usual care
UCAN: UWALK Cancer Group
